# Chronic Rhinosinusitis: Potential Role of Microbial Dysbiosis and Recommendations for Sampling Sites

**DOI:** 10.3389/fcimb.2018.00057

**Published:** 2018-02-28

**Authors:** Elizabeth Copeland, Katherine Leonard, Richard Carney, Justin Kong, Martin Forer, Yuresh Naidoo, Brian G. G. Oliver, Justin R. Seymour, Stephen Woodcock, Catherine M. Burke, Nicholas W. Stow

**Affiliations:** ^1^The School of Life Sciences, University of Technology Sydney, Sydney, NSW, Australia; ^2^Sydney Centre for Ear Nose and Throat, Frenchs Forest, Sydney, NSW, Australia; ^3^The Climate Change Cluster, University of Technology Sydney, Sydney, NSW, Australia; ^4^Department of Otorhinolaryngology, Royal North Shore Hospital, University of Sydney, Sydney, NSW, Australia; ^5^Department of Otorhinolaryngology, Concord Hospital, University of Sydney, Sydney, NSW, Australia; ^6^Woolcock Institute of Medical Research, The University of Sydney, Sydney, NSW, Australia

**Keywords:** chronic rhinosinusitis, microbiome, sinus, 16S rRNA gene sequencing, middle meatus

## Abstract

Chronic rhinosinusitis (CRS) is an inflammatory condition that affects up to 12% of the human population in developed countries. Previous studies examining the potential role of the sinus bacterial microbiota within CRS infections have found inconsistent results, possibly because of inconsistencies in sampling strategies. The aim of this study was to determine whether the sinus microbiome is altered in CRS and additionally if the middle meatus is a suitable representative site for sampling the sinus microbiome. Swab samples were collected from 12 healthy controls and 21 CRS patients, including all eight sinuses for CRS patients and between one and five sinuses for control subjects. The left and right middle meatus and nostril swabs were also collected. Significant differences in the sinus microbiomes between CRS and control samples were revealed using high-throughput 16S rRNA gene sequencing. The genus *Escherichia* was over-represented in CRS sinuses, and associations between control patients and *Corynebacterium* and *Dolosigranulum* were also identified. Comparisons of the middle meatuses between groups did not reflect these differences, and the abundance of the genus *Escherichia* was significantly lower at this location. Additionally, intra-patient variation was lower between sinuses than between sinus and middle meatus, which together with the above results suggests that the middle meatus is not an effective representative sampling site.

## Introduction

Chronic rhinosinusitis (CRS) is characterized by persistent inflammation of the nose and the paranasal sinuses with symptoms including nasal obstruction or nasal discharge, in addition to facial pain or pressure and a reduction in the sense of smell (Fokkens et al., 2012). To be distinguished from acute sinusitis, symptoms must exceed 12 weeks in duration without complete resolution. The prevalence of CRS is between 4.5 and 12% in developed countries (DeConde and Soler, 2016), resulting in substantial societal morbidity, with costs related to healthcare, lost working days (Bhattacharyya, 2009) and decreased productivity estimated to surpass $12.8 billion dollars per annum in the United States alone (DeConde and Soler, 2016).

Current medical and surgical treatments are often very broadly applied across the patient population. Maximal medical therapy (MMT) is recommended as initial treatment and may include nasal saline irrigations, topical and oral corticosteroids and antibiotics. Patients who have an inadequate response to medical treatment may be offered functional endoscopic sinus surgery (FESS), a surgical procedure aiming to restore sinus ventilation and function by widening the ostia of sinus cavities (Khalil and Nunez, 2006; Patel et al., 2017) and reducing inflammatory load (Bassiouni et al., 2012). In a quality of life study, surgical intervention in patients with high symptom scores was a more effective treatment strategy than ongoing medical treatment (Patel et al., 2017). Improved understanding of the pathophysiology of CRS should lead to more targeted treatment options.

The presence or absence of nasal polyps has been a traditional way to phenotype CRS patients into two groups; CRS with nasal polyposis (CRSwNP) and CRS without nasal polyposis (CRSsNP). There is evidence to support distinct immunologic endotypes, including T helper (Th) 1-driven pathways for CRSsNP and Th2-driven pathways for CRSwNP (Van Zele et al., 2006; Beswick et al., 2017), however, there are currently no independent methods of treatment in practice.

Originally, the sinuses were assumed to be sterile in healthy patients, but laden with bacteria in CRS patients (Meltzer et al., 2004; Ramakrishnan et al., 2013b). Early research into the pathophysiology of CRS focused on a model where inflammation was hypothesized to be driven by bacterial infection (Hoggard et al., 2017). Later, other infectious agents, including viruses and fungi, were proposed to be involved, but evidence to support these hypotheses is lacking (Boase et al., 2013; Hamilos, 2014). Non-infectious factors, including anatomic and genetic abnormalities, innate immune deficiencies, asthma, allergy, aspirin sensitivity, and biofilm formation, have all been considered as modulators of disease establishment or severity (Lam et al., 2015; Ramakrishnan and Frank, 2015; Anderson et al., 2016).

More recently, research into the pathophysiology of CRS has focused on the role of the entire microbial community residing in the sinuses (Abreu et al., 2012; Cleland et al., 2016). This is due to a shift away from culture-based methods of bacterial identification and advances in culture-independent 16S rRNA gene sequencing. With an increased understanding of interactions within microbial communities, a “dysbiosis” mechanism has been proposed as modulating inflammation in diseased sinuses (Bordin et al., 2016). The hypothesis suggests that externally influenced changes in the sinonasal microbiome can result in dysbiosis, i.e., a shift from a “normal” or “healthy” microbial community structure, and that this shift may be responsible for the initiation or maintenance of CRS (Lam et al., 2015; Jervis Bardy and Psaltis, 2016). One proposed model suggests that loss of nasal epithelial integrity allows for an increased permeability of the microbial community through the superficial layer, initiating an immune response from the host (Hoggard et al., 2017; Valera et al., 2017).

Diversity measures used in 16S rRNA gene sequencing studies can serve as useful markers of disease. For example, changes in alpha diversity, which is the diversity of species within a sample, have been observed in inflammatory airway diseases, including chronic obstructive pulmonary disease (COPD), asthma and allergic rhinitis (Ege et al., 2011; Garcia-Nuñez et al., 2014; Lynch et al., 2014). Reduced bacterial diversity in CRS patients has been reported in studies with relatively low numbers of participants (Abreu et al., 2012; Choi et al., 2014; Biswas et al., 2015), while one study reported increased bacterial diversity (Aurora et al., 2013). Other studies found no differences in alpha diversity (Cleland et al., 2016), including one of the largest studies to date with 70 CRS and 31 control participants (Ramakrishnan and Frank, 2015). As such it is still unclear whether alpha diversity is a useful marker of the CRS disease state.

Bacteria from the genera *Staphylococcus, Propionibacterium*, and *Corynebacterium* are prevalent in the sinuses of CRS patients reported across multiple studies, however, these genera are also ubiquitous in healthy subjects. No single bacterial species has been reported to be consistently higher or lower in relative abundance in CRS subjects between studies. For example, Abreu et al. found enrichment of *C. tuberculostearicum* in CRS patients (Abreu et al., 2012), while Aurora et al. found higher relative abundances of *C. accolens* (Aurora et al., 2013). Observations of depleted species in CRS include *Bacteroidetes* spp., *Prevotella* spp. (Choi et al., 2014), *Lactobacillus* spp. (Abreu et al., 2012), *Peptoniphilus* spp., *Propionibacterium acnes* (Boase et al., 2013), *Acinetobacter johnsonii*, and *Corynebacterium confusum* (Cleland et al., 2016). Differences in primers used and regions of the 16S rRNA gene targeted make it difficult to directly compare results between studies at the species level. However, in a recent meta-analysis of published 16S rRNA gene sequence data, Mackenzie et al. suggested that *Burkholderia* and *Propionibacterium* may be gatekeepers that stabilize the healthy bacterial community, as the removal of these genera from healthy datasets resulted in more fragmented networks that were potentially more susceptible to disturbance (Wagner Mackenzie et al., 2017). Still, the connection between the sinonasal microbiome and CRS has not yet been defined unequivocally (Anderson et al., 2016; Hoggard et al., 2017).

One possible reason for contrasting results in different studies is methodological differences, including variant sampling techniques and choice of sampling site. The sinuses can only be accessed through FESS (Fokkens et al., 2012) meaning there are limited opportunities to sample from the disease site, particularly in non-CRS controls. However, the middle meatus, an area of the nasal cavity that accepts drainage from the maxillary, anterior ethmoid, and frontal sinuses (Feazel et al., 2011), can be sampled without surgical intervention. As such, the middle meatus is the most commonly sampled site in studies of the sinus microbiome (Stephenson et al., 2010; Feazel et al., 2012; Aurora et al., 2013; Boase et al., 2013; Ramakrishnan et al., 2013a,b; Biswas et al., 2015; Joss et al., 2015; Kim et al., 2015; Ramakrishnan and Frank, 2015; Cleland et al., 2016; Hauser et al., 2016; Ivanchenko et al., 2016; Kaspar et al., 2016; Willis et al., 2016). One small study of eight CRS patients found the middle meatus to be broadly representative of the sinuses (Ramakrishnan et al., 2017), but this has not been explored in more detail. Furthermore, the possibility of substantial intra-patient variation, or the variation between individual sinuses within a single patient (Joss et al., 2015), is another possible confounding factor.

The primary objective of this study was to test the hypothesis that the sinonasal microbiome of CRS patients is distinctly different to those found in healthy control sinuses. We aimed to avoid confounding factors such as sampling site by sampling from as many sites as possible from each participant. This design also enabled the secondary objective of this study, which was to test the hypothesis that a swab from the middle meatus would obtain a representative sample of the entire sinus microbiome and to examine the degree of intra-patient variation across the sinuses in both CRS and control subjects.

## Materials and methods

### Patient recruitment

Study participants were recruited from the Sydney-based practices of Otorhinolaryngologists, Nicholas Stow, Justin Kong, Yuresh Naidoo, and Martin Forer, between June 2015 and January 2017. The study protocol was approved by the Northern Sydney Local Health District Human Research Ethics Committee under approval number HREC/10/HAWKE/145. Two groups of patients were informed about the study and invited to participate: firstly, those requiring endoscopic sinus surgery on all paranasal sinuses for the treatment of CRS (with or without nasal polyposis) and, secondly, controls who were undergoing sinus surgery for non-sinusitis indications and who had no clinical or radiological evidence of sinusitis. If the patient indicated interest in participating in the study, the surgeon obtained written informed consent. Diagnosis of CRS was made based on the diagnostic criteria of the EPOS guidelines (Fokkens et al., 2012). Clinical data for each patient were collected, including demographics, SNOT-22 symptom scores (Kennedy et al., 2013), asthma status, and surgical history. Patients who had taken antibiotics or oral corticosteroids in the month prior to surgery were excluded from the study.

### Sample collection

General anesthesia was administered and the nasal cavity was prepared by infiltration with a local anesthetic solution, most commonly 1% lignocaine and 1:80,000 adrenaline, then placement of pledgets soaked with topical vasoconstrictor solution, most commonly 1:2,000 adrenaline. Modern techniques of FESS, using a microdebrider (Medtronic) and through-cutting instruments (Storz), were used to preserve mucosa while creating the largest possible sinus cavities by enlargement of natural ostia. As each sinus was opened, a sterile Copan Amies Transport swab (Interpath Services) was introduced into the sinus, under endoscopic guidance. Each swab was passed through a mixing cannula, then into the nose, to protect the swab tip from contamination from other sites. Each swab was placed in a sterile Eppendorf tube and stored at −80°C until DNA extraction. For the CRS group, a total of 11 swabs were collected (from each of the following sites on the left and right side: middle meatus, maxillary sinus, ethmoid sinus, sphenoid sinus, and frontal sinus, as well as a swab from the right nostril). For the control group, swabs were collected from the right nostril, right and left middle meatus and each sinus opened at surgery. Overall, this meant the design of the study was nested such that multiple locations were sampled within each patient and each patient was nested by health status.

### DNA extraction

The genomic extraction of all swabs collected was performed using PowerSoil DNA Isolation kit (Mo Bio). Samples were removed from storage at −80°C and the swab heads were shaved, using a scalpel blade, from the metal body and prepared using an alternate method proposed by the manufacturer. This included combining the addition of solution C2 and C3 into one step and omitting the addition of solution C4. DNA was stored at −20°C in 60 μL elution buffer until further processing.

### Library preparation and sequencing

Amplification of the 16S rRNA gene V3–V4 region was performed using primers designed to anneal to the 338F and 806R positions of the *Escherichia coli* 16S rRNA gene, carried out in a two-stage protocol. Primers for PCR Stage One included the 16S rRNA gene priming regions, a 0–3 nucleotide spacer for increased diversity between sequence clusters (Wu et al., 2015) and partial Illumina sequencing adaptors (Table S1). PCR Stage Two primers contained an overlap of the adaptor region from PCR Stage One, a sample barcode (8 nt) and a flow cell adaptor region compatible with the Illumina MiSeq (Table S2).

All amplifications were performed in 50 μL reactions using Taq Core PCR Kit (Qiagen); 1X buffer, dNTPs at 250 μM, 1.25 U Taq polymerase, and 0.5 μM each of both forward and reverse primers (Table S1). The first amplification step was performed under the following thermal cycling conditions: one initial denaturation step at 95°C for 3 min; 20 cycles at 95°C for 15 s, at 55°C for 30 s and at 72°C for 40 s; and extension at 72°C for 3 min; and a hold step at 4°C. A cleanup procedure was performed on the resultant amplicons (>200 bp) using a 0.8X volume of magnetic AMPure XP beads (Beckman Coulter) to purify the amplicons from free primers and primer dimer species, following the manufacturer's specifications.

In the second amplification step, the maximum amount of the purified product of the first PCR (27.5 μL) was used as template in a reaction which included 0.5 μM each of the enrichment_i7 and enrichment_i5 primers (see Table S2) in a total volume of 50 μL. Unique combinations of indexes from i7 and i5 enrichment primers were used for sample barcoding. Reactions were subject to thermal cycling of one initial denaturation step at 95°C for 3 min; 15 cycles at 95°C for 15 s, at 55°C for 30 s and at 72°C for 60 s; and extension at 72°C for 3 min; and a hold step at 4°C. Positive (*E. coli* DNA) and negative controls were included with every PCR run. The ZymoBIOMICS microbial community DNA standard (Zymo Research) (hereafter called the mock community) consisting of eight bacterial and two yeast species was amplified as a sequencing control to assess consistency across sequencing runs, and to ensure that the PCR and sequencing conditions used were able to accurately capture a community of known composition. A cleanup procedure on the resulting amplicons was performed as described above and the final DNA concentrations of the purified products were assayed using the Qubit Fluorometer 2.0 (Thermo Fisher Scientific) as per the manufacturer's instructions. Samples were combined into two pools with approximately equal concentrations of 16S rRNA gene amplicons from each sample, with the mock community and PCR negative controls included in each sequencing run, and underwent a final cleanup procedure as described above to obtain a final concentration of approximately 5 nM. Both libraries were sequenced on an Illumina MiSeq using the Reagent Kit V3 with 600 cycles (Illumina). Libraries were denatured and diluted according to the manufacturer's recommendations and loaded at a concentration of 12 pM with 5% PhiX control.

### Sequence analysis

Sequences were demultiplexed with Phylosift 1.0.1 (Darling et al., 2014) and merged using FLASh 1.2.11 (Magoc and Salzberg, 2011) with default settings, except for a minimum overlap of 80 and maximum of 140 bases. Merged sequences were quality filtered using the fastq_filter command in USEARCH v9.0.2132 (Edgar and Flyvbjerg, 2015) with the fastq_maxee setting at 2 to remove sequences with more than 1 expected error.

The mock community sequences only were analyzed using BLAST 2.6.0 (Altschul et al., 1997) against a local database of the known community sequences. Only the best hit was retrieved for each sequence and the identity of the best hit was used to assign each sequence to a member of the mock community. The relative proportion of each species obtained in each sequencing run were compared to the expected proportions from the DNA mixture and the log_2_ fold-change from the expected relative abundance was calculated.

The QIIME 1.9.1 software package (Caporaso et al., 2010) was then used for Operational Taxonomic Unit (OTU) picking, using the pick_open_reference_otus.py workflow script. Chimeric sequences were identified using the identify_chimeric_seqs.py command with the usearch61 method and removed with the filter_otus_from_otu_table.py script. Taxonomy was assigned using the SINA 1.3.1 alignment and classification tool (Pruesse et al., 2012) with the SILVA REF NR 99 ssu database (released 13th December 2017), accessed from the SILVA website^1^.

The data were rarefied to 6,000 sequences per sample for all downstream analyses using the single_rarefaction.py script. The OTU table was filtered to include only OTUs that were present in at least 5% (*n* = 12) of samples during determination of differential abundance of OTUs and taxonomic groups between groups of samples. For tests of differential abundance or correlation to metadata, in order to account for repeated sampling from individuals counts of OTUs or taxa from samples from the same individual were summed, followed by normalization of total counts per individual to 1 using the collapse_samples.py script. For tests at higher taxonomic levels, the OTU table was collapsed per taxonomic level, followed by collapsing counts per individual as described above. Tests for differential abundance (by health status, nasal polyp status within the CRS group and by sinus within the control and CRS groups) were performed at each level using the group_significance.py script and the default Kruskal-Wallis test with Benjamini-Hochberg FDR correction to account for multiple testing. Tests were performed on sinus samples and middle meatus samples separately. Correlation of the OTU relative abundance level and SNOT-22 scores was performed using the observation_metadata_correlation.py script. Further analyses were then carried out in R 3.4.0 (Ihaka and Gentleman, 1996). The alpha diversity metric employed (Shannon) considers both the number and the distribution of species (Shannon) (Shannon, 1948) at a local site. Weighted Unifrac (Lozupone and Knight, 2005) was used as the beta diversity metric to compare overall similarities or dissimilarities of the whole microbial community structure between local sites. Alpha diversity, beta diversity, and Principal Coordinates Analysis (PCoA) were calculated with the Phyloseq 1.20.0 (McMurdie and Holmes, 2013) and Vegan 2.4 (Oksanen et al., 2017) packages. Plots were produced using the dplyR 0.7.0 (Wickham et al., 2017) and ggplot2 2.2.1 (Wickham, 2016) packages. For comparisons of alpha diversity, statistical analyses were performed using the lme4 1.1 (Bates et al., 2015) and lemrTest 2.0 (Kuznetsova et al., 2016) R packages and the ANOVA function, with a model specifying repeated measures from individual patients as a random variable. PERMANOVA, implemented in the adonis function of the Vegan package, was used to determine differences in beta diversity between groups of interest. Tests for homogeneity of group dispersions and differences between groups were carried out with the betadisper and permutest functions in the Vegan package. The intra-patient weighted and unweighted unifrac distances between the sphenoid and middle meatus of the healthy group and the CRS group and the intra-patient distances comparing the middle meatus samples with nostril and sinuses were tested using ANOVA. For all statistical tests, significance was defined as *p* < 0.05. Designation of genera as anaerobic was done manually based on literature searches. All genera known to be anaerobic or facultative anaerobic were selected and the relative abundances plotted.

In order to examine potential host to microbe and microbe to microbe interactions which may influence the disease state, network analysis was performed using the extended local similarity analysis (eLSA) pipeline (Xia et al., 2011). eLSA enabled identification of statistically significant patterns in the co-occurrence of bacterial OTUs, alpha diversity, and disease state across sinus samples from 32 patients. Thereby, teasing out from a vast background of correlations, the interactions that most likely represent relationships that are meaningful within a disease context. Each sinus sample was designated as a replicate within its respective patient and missing values (representing 0.4% of the dataset) were linearly interpolated using the *linear fill missing* function as employed by Needham et al. (2013). Filtering criteria included removal of pairwise interactions that occurred across < 10% of the sample replicates, and each remaining interaction was subjected to 500 permutations to estimate *p*-values (the probability of a false positive) and q values (the probability of a false negative). Interactions where *p* ≥ 0.001 and *q* ≥ 0.01 were excluded to reduce the risk of Type I and Type II errors. Resultant data included all statistically significant co-occurrence values, which was prepared for visualization by first assigning local Similarity (*LS*) values of pairwise interactions to edges, then plotted into a network using the edge-weighted spring embedded function in Cytoscape 3.0 software package (Shannon et al., 2003).

## Results

A total of 31 subjects were included in the study, including 12 healthy controls and 21 CRS patients. 57% (*n* = 12) of CRS patients had nasal polyps. Patient characteristics were collected and are displayed in Table S3, with a total of 287 swabs collected (indicated in Table S4).

### Sequencing data

Quality filtered sequence data from the two sequencing runs was deposited in the European Nucleotide Archive under accession numbers ERR2145113 and ERR2145112. Additionally, data is available on the Qiita website^2^ under study ID 11622. A total of 18,377,078 sequences passed quality filtering steps and chimera removal across 292 samples, with a median coverage of 45,911 sequences per sample. Analysis of the mock community sequences from the two sequencing runs showed that 98% of sequences passing quality filters were within 97% similarity to the reference sequences from the mock community. Across the two sequencing runs, similar abundances were achieved for the mock community members, all within −0.5 to 0.5 log_2_ fold change from the expected abundance (Figure S1). Coverage of three PCR and DNA extraction process negative controls was low (329, 542, and 3,255 sequences after quality filtering) and these sequences were dominated by the common skin bacterial genera *Corynebacterium* and *Staphylococcus*. Given that these genera are also likely to be in sinus and nasal samples, we did not filter any taxa from the data. Instead we required sequence coverage of more than or equal to 6,000 sequences for inclusion in the sample data. Based on rarefaction analysis, all samples were rarefied to 6,000 sequences per sample for further analysis.

### Comparison of sinus microbial communities between CRS and controls

**Figure 1:**
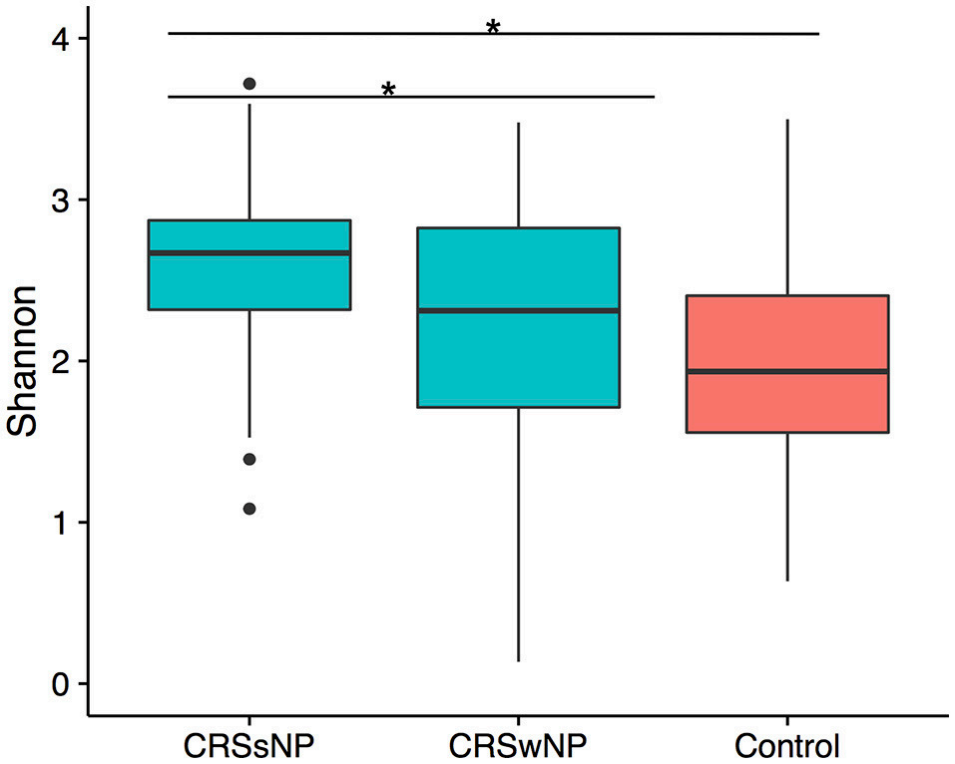
Boxplot of Shannon diversity. **(A)** Diversity in sinus cavities from CRS subjects separated by the absence (CRSsNP) or presence (CRSwNP) of nasal polyps and control subjects. Significant differences indicated by ^*^ (ANOVA, *p* < 0.05).

To determine if alpha diversity is a suitable marker of disease, differences in Shannon diversity were examined between the control and the disease group. When considering sinus cavities only, there was no significant difference (ANOVA; *p* > 0.05), however, when the CRS group was split into CRSwNP and CRSsNP, both control and CRSwNP sinuses had significantly lower diversity than CRSsNP (Figure 1).

**Figure 2:**
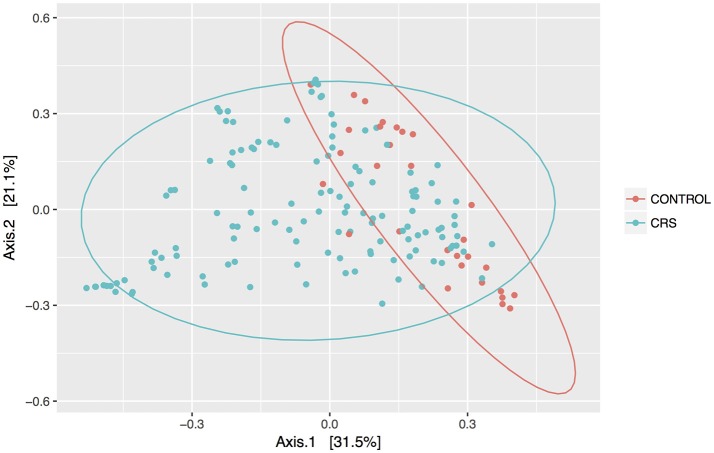
PCoA of weighted Unifrac distances. Samples from sinus cavities only are included, and ellipses represent 95% confidence intervals.

Discrimination of total control and CRS communities was explored through beta diversity analyses. Pairwise distances between samples were calculated using the weighted unifrac metric and visualized as PCoA plots (Figure 2). CRS samples overlapped substantially with controls, but a significant PERMANOVA result suggested that the communities differed between control and CRS sinuses. The size of this effect was indicated by the *R*^2^-value of the test, which showed that health status (i.e., CRS vs. controls) accounted for 7% of the variation in the dataset (*p* < 0.001), while the largest amount (51.5%) was due to inter-individual variation (*p* < 0.001). CRS samples visually appeared more dispersed, which can result in a false significant PERMANOVA score. However group dispersion was not significantly different between CRS and controls (betadisp test *p* > 0.05), indicating that the significant result was due to a difference in community structure, as opposed to dispersion. A significant difference in beta diversity between CRS subtypes CRSwNP and CRSsNP was detected (PERMANOVA *p* < 0.001), however in this case the beta-dispersion was significantly different between groups.

### Differentially abundant microbes in CRS are correlated with disease state and diversity

**Figure 3:**
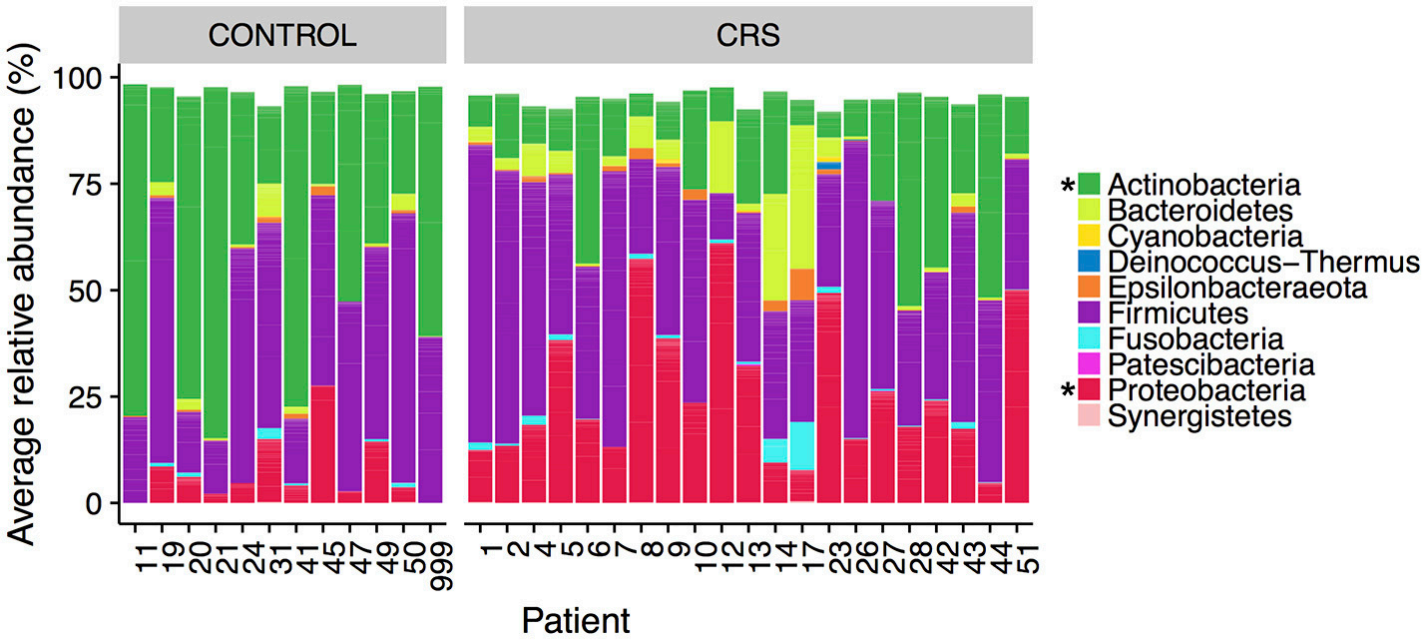
Average relative abundance of phyla in CRS and control subjects. Values are averages across all sinus samples per patient. Percentages were calculated from rarefied data. Phyla that were significantly different in relative abundance between CRS and controls are marked with ^*^ (Kruskal-Wallis, FDR corrected *p* < 0.05).

**Figure 4:**
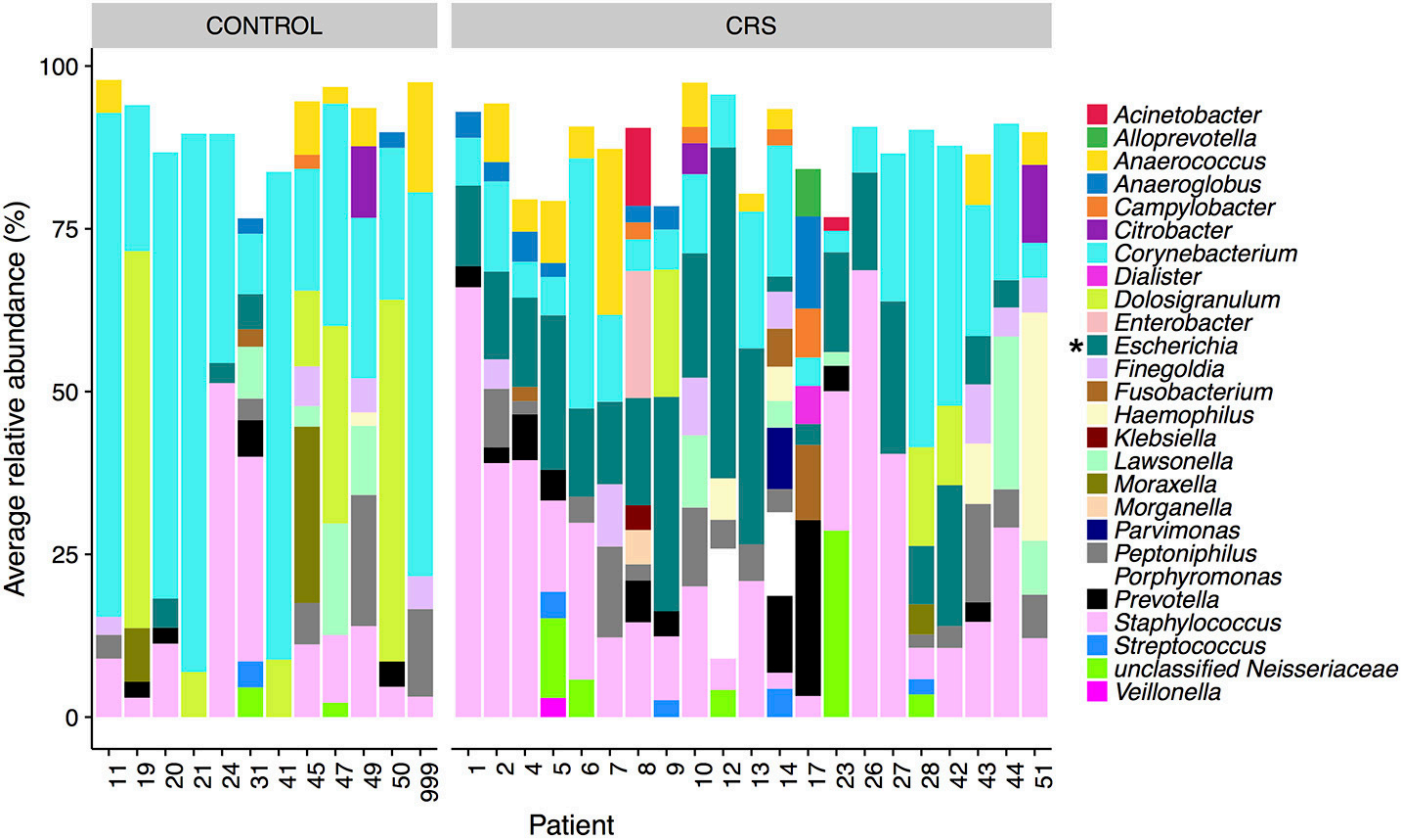
Average relative abundance of OTUs per patient colored by genus. Only sinus samples were included, and OTUs with average relative abundance of <1% were excluded. Percentages were calculated from rarefied data. Genera that were significantly different in relative abundance between CRS and controls are marked with ^*^ (Kruskal-Wallis, FDR corrected *p* < 0.05).

The microbial communities in the sinuses of all subjects were represented primarily by the phyla Firmicutes, Actinobacteria, Proteobacteria, and Bacteroidetes. In the CRS group, Proteobacteria were significantly more abundant overall (Kruskal Wallis, FDR adjusted *p* < 0.01) (See Figure 3). At the level of genus, only *Escherichia* was significantly different with higher relative abundance in CRS (Kruskal-Wallis test with FDR adjusted *p*-value < 0.01) (Figure 4). This was reflected at the OTU level, where five OTUs classified as *Escherichia* were significantly more abundant in the CRS group (Kruskal-Wallis test with FDR adjusted *p*-values < 0.05). We note that the taxonomic assignment according to the SILVA taxonomy is listed as *Escherichia-Shigella*. For the sake of simplicity, we refer to this genus simply as *Escherichia*.

In the control group, the Actinobacteria were significantly more abundant (Kruskal Wallis, FDR adjusted *p* < 0.01). No genera were significantly more abundant in the controls, however one OTU classified as *Corynebacterium* (OTU 410908) was. When comparing CRSwNP to CRSsNP, no OTUs or taxa were significantly different.

**Figure 5:**
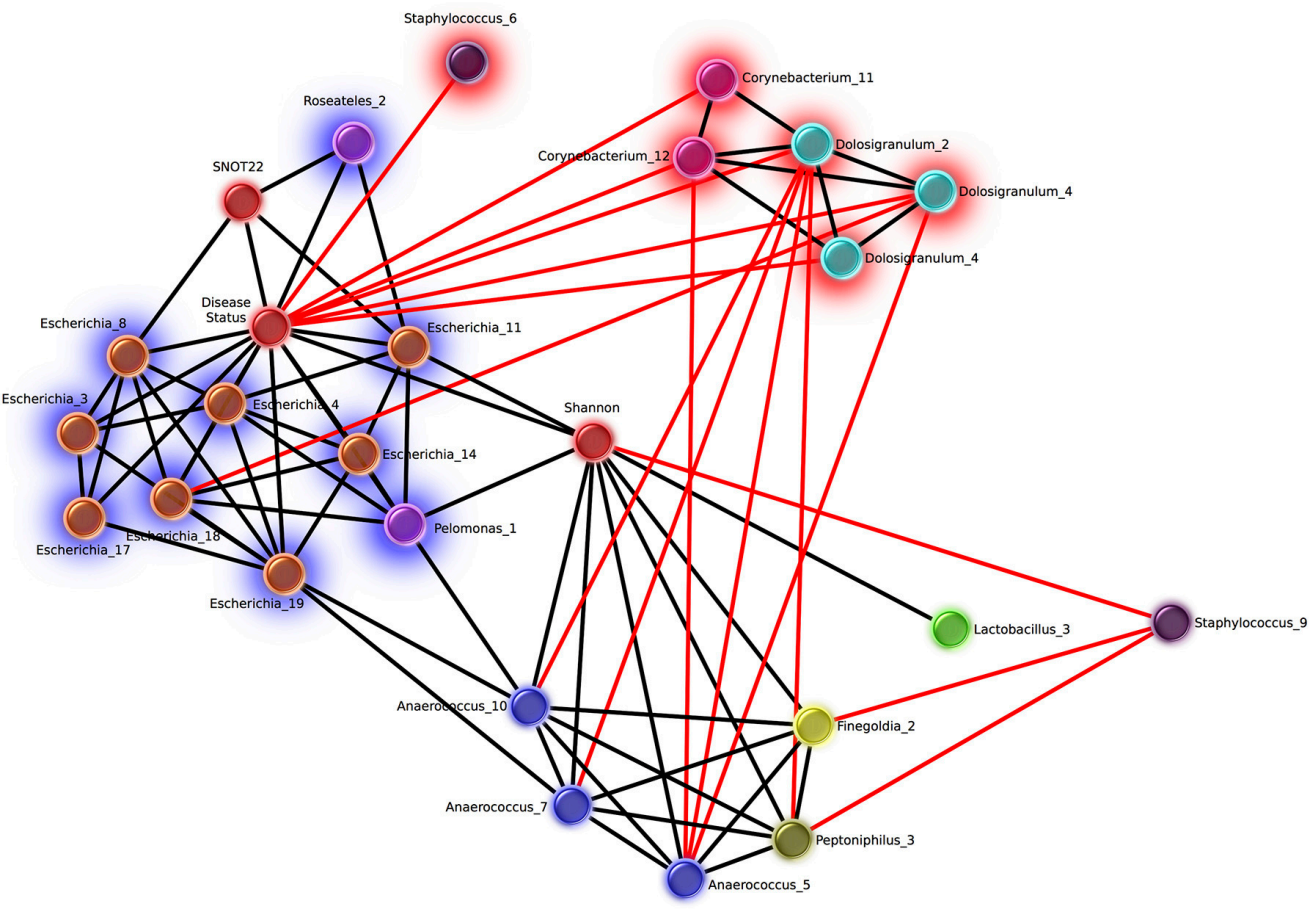
Network of OTUs associated with disease status, and strong significant correlations to other OTUs. Networks were calculated using extended local similarity analysis and plotted using the Cytoscape3 software package. Nodes are colored by genus taxonomic assignment, and metadata category nodes are colored red. Glow behind nodes indicates the relationship to disease status (blue, positive correlation; red, negative). Black lines indicate positive correlations, and the length indicates the strength (short, strong; long, weak). Red lines indicate negative correlations and the length indicates the strength in the opposite direction to black (long, strong; short, weak).

Network analysis revealed 16 OTUs with significant eLSA scores for correlation to disease status (Figure 5). The abundance of six OTUs were negatively correlated with the CRS disease state, three of which were *Dolosigranulum*, two were *Corynebacterium*, and one *Staphylococcus*. *Corynebacterium* and *Dolosigranulum* OTUs were positively correlated with each other. The 10 OTUs positively correlated with CRS consisted of eight OTUs classified as *Escherichia* and two OTUs from the Burkholderiaceae family (genera *Roseateles* and *Pelomonas*), which were all positively correlated with each other. OTUs from the genera *Corynebacterium* and *Escherichia* were also identified in the differential abundance results described above (from Kruskal Wallis tests), however only five OTUs (all *Escherichia*) were concordant across the two analyses.

Of the other categories included in the network analysis, including age, sex, smoking status, and history of asthma, only SNOT-22 scores and Shannon Diversity correlated with disease. Disease status was positively correlated with Shannon diversity, which was in turn positively correlated with several OTUs from taxa that are strict and facultative anaerobes, including *Finegoldia, Anaerococcus, Peptoniphilus*, and *Lactobacillus*. Smoking status was negatively correlated with 6 *Staphylococcus* OTUs and positively correlated with 10 OTUs classified as *Fusobacteria* (*n* = 2), *Prevotella* (*n* = 4), *Dialister* (*n* = 2), *Campylobacter* (*n* = 1), *Slakia* (*n* = 1), *Escherichia* (*n* = 1), and *Citrobacter* (*n* = 1) (Figure S2).

Strict and facultative anaerobic genera (designated as such via manual literature searches) that accounted for >1% average relative abundance per patient included *Anaerococcus, Dialister, Finegoldia, Porphyromonas, Parvimonas*, and *Prevotella*. CRS patients 7, 10, 14, 17, and 43 had high levels of these anaerobic genera (between 10 and 80% relative abundance) across all or most sinuses that yielded data. Most other CRS patients had high levels in one or more sinuses, with the exception of patients 26, 27, 28, and 42 (Figure S3). Half of the control patients had low (<10%) relative abundance of these anaerobic genera in all sinuses sampled (Figure S4).

### Correlation of bacterial taxa to symptom severity

To determine whether the abundance of particular bacterial taxa were associated with increased or decreased severity of disease symptoms, Spearman's correlations were calculated for both OTUs and genera against SNOT-22 scores. SNOT-22 scores range from 0 to 110, with higher scores associated with more severe symptoms. Nineteen OTUs had significant correlations (FDR corrected *p* < 0.05) to SNOT-22 scores, with the strength of correlations ranging from −0.52 to 0.61.

**Figure 6:**
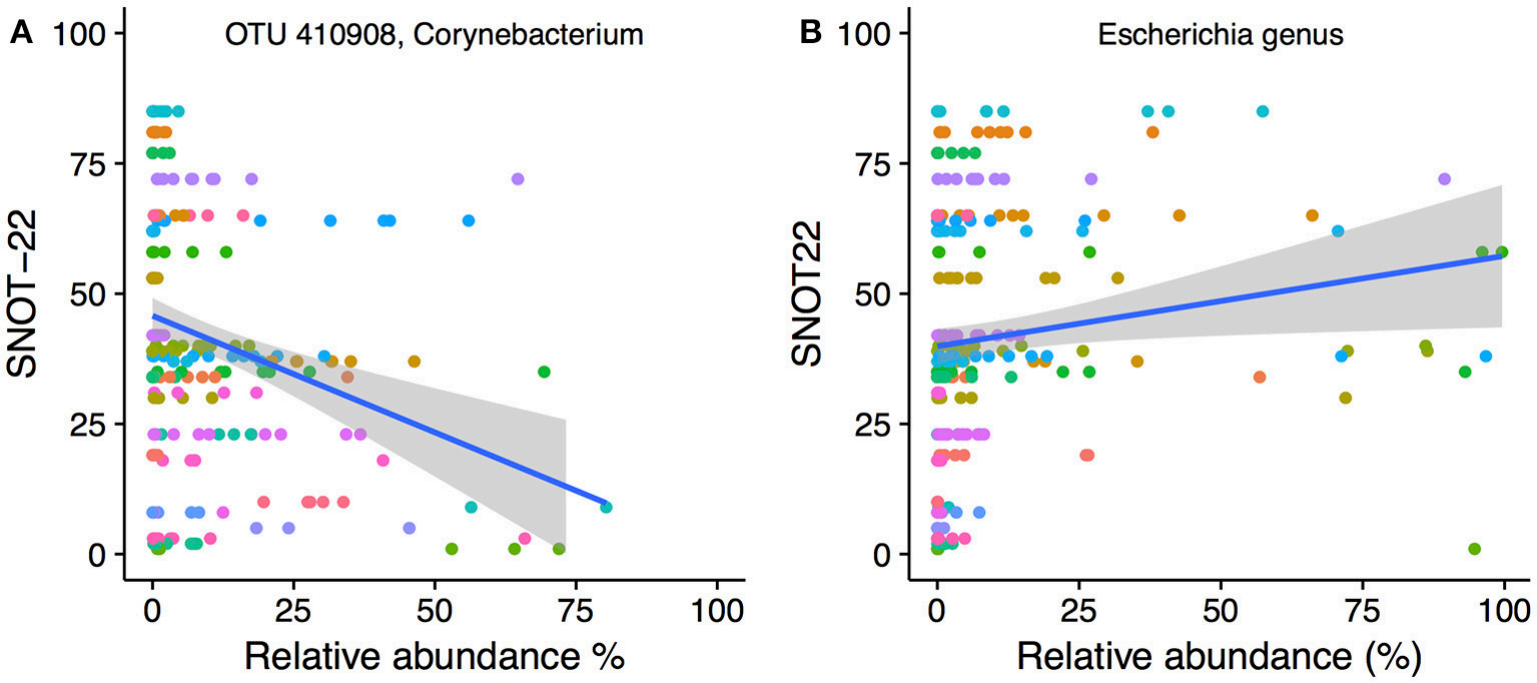
SNOT-22 vs. relative abundance for **(A)** one *Corynebacterium* OTU with a negative correlation, and **(B)** the genus *Escherichia* with a positive correlation. Plots are overlaid with a linear regression line and shading indicating 95% confidence interval. Individual patient samples are indicated with color. Rarefied data were used to generate the plot.

Only one OTU (OTU 410908, *Corynebacterium*) was negatively correlated to SNOT-22 scores. A scatter plot with linear regression line overlaid is shown in Figure 6A. No genera were negatively correlated to SNOT22 scores. Positive correlations to SNOT-22 scores were found for 18 OTUs, nine of which were assigned to *Escherichia* (*n* = 9), The other nine OTUs were present at very low relative abundance and only showed higher relative abundance in a limited number of samples (see Figure S5 for plots of every OTU with a significant correlation). At the genus level, only *Escherichia* was positively correlated (0.61), as shown in Figure 6B.

### Is the middle meatus representative of sinus microbial communities?

**Figure 7:**
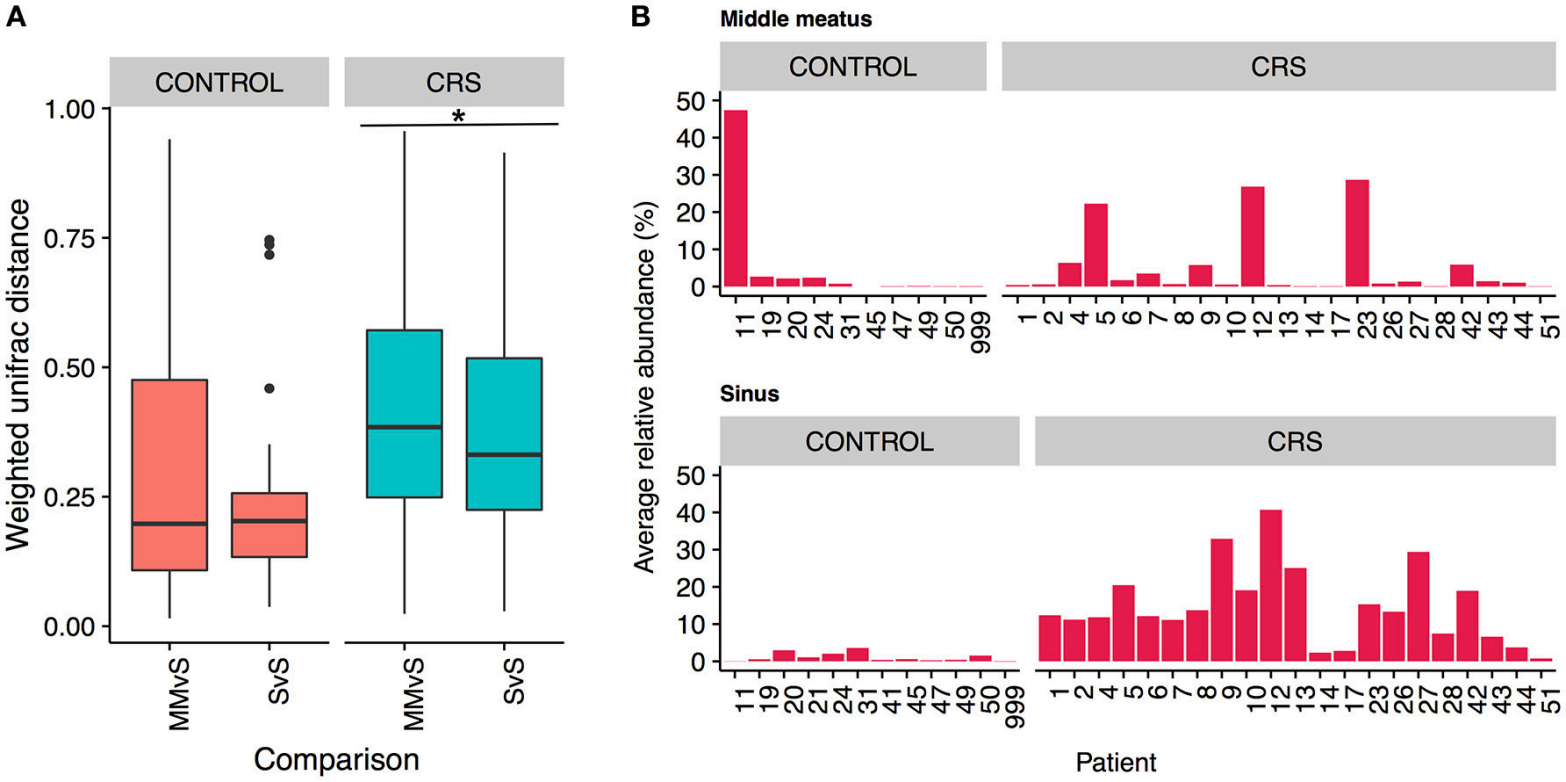
Differences between middle meatus and sinus microbial communities. **(A)** Intra-patient distances between middle meatus and sinus (MMvS) and sinus and sinus (SvS) samples for weighted Unifrac distances. Significant difference is indicated with ^*^ (ANOVA, *p* < 0.05). **(B)** Average relative abundance of the genus *Escherichia* in middle meatus samples (top panels), and sinus samples (bottom panels).

To determine whether the more easily accessible middle meatus samples were representative of an individual's sinuses, we calculated intra-patient weighted unifrac distances for middle meatus vs. sinuses (MMvS) and compared those to sinuses vs. sinuses distances (SvS) (Figure 7A). CRS patients had a small but significant difference between MMvS and SvS distances, where sinuses were more similar to each other than the middle meatus was to the sinuses.

To explore this further, we looked for genera that were consistently differentially abundant between middle meatus and sinus samples. No genera were identified in control patients, while in CRS patients *Escherichia* was significantly lower in the middle meatus (Kruskal-Wallis test with FDR adjusted *p*-value < 0.01). The average relative abundance of *Escherichia* in sinuses and middle meatus samples is shown in Figure 7B. Tests at the OTU and genus level for differential abundance between CRS and control groups on middle meatus samples only did not detect any significant differences.

**Figure 8:**
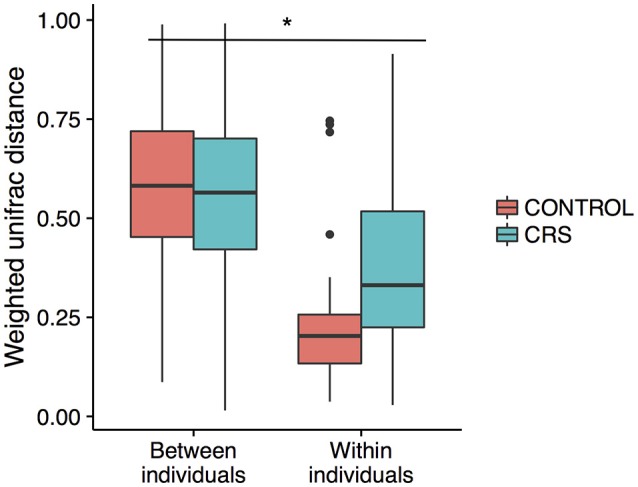
Intra vs. inter-individual weighted Unifrac distances in CRS and control subjects. Inter-individual distances were significantly higher than intra-individual distances, indicated with ^*^(ANOVA, *p* < 0.05). A trend of higher intra-individual distances in CRS subjects was observed, but only approached significance (ANOVA, *p* = 0.06).

Additionally, the intra-patient distances were compared between the control and CRS groups to determine whether CRS is associated with more disparate microbial communities between sites within an individual. A trend of higher between site intra-patient distances (weighted unifrac) was observed between microbial communities within the same individual in CRS compared to controls (Figure 8), which approached but did not reach the significance cut-off (*p* = 0.06). Inter-patient variability was significantly higher than intra-patient variability (*p* < 0.0001).

## Discussion

The concept of “dysbiosis” in the sinus cavities during CRS has gained traction due to observations of shifts in bacterial community structures, delivered by 16S rRNA sequencing technologies (Bordin et al., 2016). However, several methodological limitations, including substantial variation in the native microbiome between individuals, limitations in obtaining larger samples sizes for control subjects and the influence of other disease-modulators, have hindered efforts to confirm this hypothesis (Anderson et al., 2016; Lee et al., 2016). This is further compounded by the known issues of differences in primer bias and regions of the 16S rRNA gene analyzed across studies. Additionally, the existence of spatial variation of the microbiome between sinus cavities has not yet been adequately addressed, which has implications for optimal microbiome sampling in CRS and sinus microbiome research.

### CRS was associated with changes in some diversity measures

A number of diseases have been shown to correlate with increased (Liu et al., 2013) or decreased (Kostic et al., 2015) microbiome diversity, indicating that a change in alpha diversity can be a useful marker of the disease state. Whether alpha diversity is a marker of CRS is unclear from previous studies (Abreu et al., 2012; Aurora et al., 2013; Choi et al., 2014; Biswas et al., 2015; Ramakrishnan and Frank, 2015; Cleland et al., 2016). We saw significant trend of higher alpha diversity in CRSsNP compared to controls in the sinus cavities. We also saw a significant difference between CRS subtypes, however given that the large overlap between these groups the biological significance of this finding is unclear. Network analysis provided further evidence for an increase in diversity and/or evenness in the disease state, with a significant positive correlation between Shannon diversity and CRS (Figure 5). Given the disparity in results between our own and previous studies, it is likely that larger sample sizes and consistency of sampling site will be needed to definitively determine whether alpha diversity is an indicator of disease state in CRS.

Weighted unifrac was used as the beta diversity metric to compare the microbial community structure between samples. PERMANOVA analysis indicated that disease status (CRS or control) was a significant factor, suggesting that the community profiles differ and providing further evidence to support the hypothesis that a shift in sinus microbial communities is associated with CRS (Stephenson et al., 2010; Biswas et al., 2015; Ramakrishnan et al., 2015; Cleland et al., 2016; Cope et al., 2017). Studies of the microbiome in other sites of the airways have similarly found microbial community shifts associated with disease. For example, infectious exacerbations of COPD lead to changes in the bacterial microbiome, including increases in *Moraxella* spp. (Wang et al., 2016; Wilkinson et al., 2017).

### CRS was correlated with the presence and abundance of specific bacterial taxa

In this study, a higher relative abundance of one *Corynebacterium* OTU was consistently found in healthy sinuses, when comparing to the CRS sinuses. Network analysis showed that two other *Corynebacterium* OTU were correlated to a reduced SNOT-22 score, in addition to directly negatively correlated to CRS disease. This is consistent with a recent survey of postoperative success in CRS subjects undergoing FESS, which found an inverse relationship between *Corynebacterium* and SNOT-22 score (Jain et al., 2017), supporting a possible probiotic nature of some species within the genus. Other observations in previous studies have also shown higher abundance of the genus *Corynebacterium* in controls compared to CRS. For example, Cleland et al. found that *C. confusum* was correlated to healthy individuals (Cleland et al., 2016). Not all members of the genus may show the same interaction, as *C. accolens* and *C. tuberculostearicum* have been shown to be enriched in CRS sinuses (Abreu et al., 2012; Aurora et al., 2013).

The abundance of three *Dolosigranulum* OTUs were negatively correlated to the CRS health state. This genus is dominant in nasal communities (Biswas et al., 2015) and the middle meatus samples of healthy patients (Ramakrishnan et al., 2013b), while also present at low abundance in the middle meatus of CRS patients (Kim et al., 2015). *Dolosigranulum* has been found to co-colonize with *Corynebacterium* species, including *C. propinquum* (Kaspar et al., 2016), and Yan et al. reported OTUs assigned to *Dolosigranulum* were top predictors of *Staphylococcus aureus* carriage (Yan et al., 2013). These genera have been reported in other areas of the upper airways, correlated with a decreased risk of *Streptococcus pneumoniae* colonization in the upper airways (Pettigrew et al., 2012) and acute ear infections in children that had no reported antibiotic use for the previous 6 months (Laufer et al., 2011).

*Propionibacterium* are widely detected in the sinuses in both healthy and CRS subjects (Feazel et al., 2012; Aurora et al., 2013; Boase et al., 2013), and were identified as a possible “Gatekeeper” taxa in healthy sinuses (Wagner Mackenzie et al., 2017). We did not detect *Propionibacterium* here, most likely because of a known mismatch with the penultimate base in the reverse primer with the 16S rRNA gene in *P. acnes* (Gohl et al., 2016). We did attempt to create our 16S rRNA gene sequencing libraries using a polymerase (Kapa Hifi, Kapa Biosciences) which has been shown to be capable of editing this base and capturing the genus *Propionibacterium* (Gohl et al., 2016). However, for reasons we were unable to discern, we were unable to amplify any of our samples with this particular polymerase.

*Staphylococcus* has been commonly implicated in CRS pathology (Choi et al., 2014; Biswas et al., 2015; Ramakrishnan et al., 2015; Cleland et al., 2016). However, this genus encompasses multiple species, including *Staphylococcus epidermidis* and *S. aureus*, which have been associated with healthy individuals and those with CRS, respectively (Stephenson et al., 2010; Feazel et al., 2012; Boase et al., 2013). Since it is difficult to accurately classify OTUs to the species level based on fragments of the 16S rRNA gene, it is challenging to draw specific conclusions from a relative increase or decrease in this genus. In this study, *Staphylococcus* did not correlate to disease severity and was not significantly higher in relative abundance in the disease group: in fact, one OTU was negatively correlated to disease. Another *Staphylococcus* OTU was negatively correlated to Shannon diversity (which was positively correlated to disease) and additionally negatively correlated to CRS-associated genera (Figure 5), suggesting a potential benefit of some *Staphylococcus* spp. in the healthy sinus cavity.

The “health-associated” taxa identified here could provide a protective environment against pathogens either passively, through competition for space or resources, or actively through secretion of antimicrobial compounds (Psaltis and Wormald, 2017). Alternatively, these genera could be the most susceptible to changes that occur in the sinus environment as a result of the CRS disease process.

Members of the genus *Anaerococcus* exhibited positive correlations to Shannon diversity (Figure 5) which was positively correlated to the disease state. *Anaerococcus*, along with *Finegoldia* and *Peptinophillus* (also correlated to increased Shannon diversity) are anaerobic. Anaerobic genera overall were observed to be more prevalent in CRS including those mentioned above and *Dialister, Porphyromonas, Parvimonas*, and *Prevotella*. Anaerobic taxa such as *Peptoniphilus, Anaerococcus*, and *Prevotella* have been reported as abundant taxa in CRS (Stephenson et al., 2010; Bassiouni et al., 2015; Biswas et al., 2015; Joss et al., 2015; Kim et al., 2015; Cleland et al., 2016; Ivanchenko et al., 2016). Conditions within the sinus cavities are not usually anaerobic, and the expansion of anaerobic bacteria in CRS may be indicative of environmental changes to the sinuses as a result of disease pathology (Brook, 2006).

In our study, the genus *Escherichia* was significantly more abundant across CRS patients when compared to controls and also positively correlated to increased symptom severity scores. Network analyses further confirmed the direct and strongly positive correlation of several *Escherichia* OTUs to CRS (Figure 5). The link between an inflammatory environment and subsequent proliferation of *E. coli* has been observed in the gut, where the release of reactive nitrogen species as an immune response can be utilized by these organisms for cellular respiration and growth (Scales et al., 2016). Thus, an increase in relative abundance of *Escherichia* in CRS may be related to the characteristic inflammation of the sinuses, and may result in exacerbation of the inflammatory host response.

### Microbial communities in CRS with and without nasal polyps

The presence of nasal polyps was associated with lower Shannon diversity when compared to CRSsNP sinuses, however no other significant differences were found in terms of bacterial community composition. A previous study found no differences in community structure between disease sub-types (Ramakrishnan et al., 2015), while a more recent study found a higher relative risk of nasal polyposis associated with microbial communities dominated by the Corynebacteriaceae family (Cope et al., 2017). So far, there is not a strong indication from the literature that these different CRS subtypes are associated with different microbial communities.

### Reflections on dysbiosis in CRS

While characterization of the CRS microbiome has led to inconsistent findings between studies, the dysbiosis hypothesis has been widely suggested as a mechanism involved in CRS pathogenesis (Ramakrishnan and Frank, 2015; Anderson et al., 2016). Dysbiosis occurs following a breakdown of the network of bacteria, leading to community-wide alterations in the microbiota (Petersen and Round, 2014; Jervis Bardy and Psaltis, 2016; Wagner Mackenzie et al., 2017). This could include “keystone species,” or microbes that normally maintain a stable and interactive community, cohabitating with a consortium of low abundance bacteria in the healthy state (Wagner Mackenzie et al., 2017). The removal of these key species may have a significant effect on the community, for example, by allowing the overgrowth of potentially pathogenic species.

Our study supports the idea of bacterial community collapse in CRS. We identified different potentially “health-associated” OTUs within the genera *Dolosigranulum* and *Corynebacterium*. Further investigation is required to elucidate any influence on health, including a possible role in increased resilience of the community, and direct interactions with the host immune system. In accordance with the dysbiosis hypothesis, the depletion or reduction of these species may allow for the growth of normally rare taxa that promote or contribute to a prolonged inflammatory state, for example bacteria from the genus *Escherichia* (Steimle et al., 2016). Conversely, changes in “health-associated” taxa may occur as a secondary effect; that these taxa may only be sensitive to the prolonged inflammatory state, or sensitive to changes in potential opportunistic pathogenic species. The directionality of this effect cannot be determined from this study. Caution should also be applied before identifying particular organisms as “pathogenic,” as their presence alone does not necessarily indicate disease. The increased abundance of particular taxa in CRS or with disease severity may be due to the loss of community structure, for example, antagonism with keystone species or loss of major network interactions between bacterial members.

The role of the eukaryotic community was not explored in this study. Fungi have been observed via culturing and molecular methods in previous studies, however no significant differences in the richness or prevalence of fungus between CRS and controls has been observed so far (Boase et al., 2013; Cleland et al., 2014a; Zhao et al., 2018). Other potential mediators of disease which were not explored in this study include the viral community (including bacteriophage) (Lee et al., 2015; Rowan et al., 2015) and the host response (Lam et al., 2015).

While changes in CRS communities have been confirmed, it is unknown whether these changes are sufficient to initiate CRS, to exacerbate or prolong an inflammatory state, or whether they exist as a non-deleterious consequence of the disease (Hoggard et al., 2017). Part of the dysbiosis mechanism may involve loss of the Sino-nasal epithelial layer integrity, with bacteria (or other pathogens) and their metabolites activating the immune system, further aggravating and prolonging inflammation (Bordin et al., 2016). The future use of animal models to explore the influence of both disease and health associated organisms identified here could be an effective means to establish potential functional roles in chronic sinus disease. Additionally, investigation into the potential for reduction in symptom severity by the inoculation of health-associated taxa could provide a viable alternative to the current ineffective use of antibiotics (Cleland et al., 2014b).

### Variation within the sinus cavities and potential for sampling error in the middle meatus

The large effects of inter-individual variation in microbial communities have been well documented in CRS studies (Biswas et al., 2015; Joss et al., 2015; Kim et al., 2015; Ramakrishnan et al., 2017), in healthy individuals (Kaspar et al., 2016) as well as in microbiome studies of other body sites (Huttenhower et al., 2014). In this study, inter-subject differences explained the largest proportion of variation within the bacterial communities (Figure 8).

Comparison of the variation in microbial communities across sinuses within an individual has been limited to studies without a healthy group. Joss et al. determined the existence of substantial variation in some individuals with CRS (Joss et al., 2015), and Ramakrishnan et al. found varying levels of similarity when comparing communities at genus level (Ramakrishnan et al., 2017). Our study design is unique in that multiple sinuses in both CRS and control groups were sampled, enabling examination of the intra-patient variation in both CRS and control groups. As expected, intra-patient variation was significantly lower than inter-patient variation and there was a trend of higher intra-patient variability in the CRS group. This result only approached significance, potentially because of the comparatively low number of comparisons in the control group. If this trend is real, it suggests that the microbial communities in the sinuses within an individual diverge during CRS, which has implications for selecting a representative sampling site.

Due to the increased accessibility of the middle meatus in comparison to the sinus sites, it is important to consider if sampling from this site will obtain a representative snapshot of the resident bacteria. Within an individual, the bacterial communities from sinuses were significantly more similar to each other than they were to communities from the middle meatus, albeit a small difference (Figure 7A). Importantly, the differences observed in the sinuses at both OTU and genus level with health status were not reflected in the corresponding middle meatal samples. Specifically, middle meatal samples underestimated the relative abundance of the genus *Escherichia* (Figure 7B), which as demonstrated here may have an important association with CRS. While broadly similar in taxonomic composition, the middle meatus does show differences to the sinus microbial communities, as has been seen previously (Ramakrishnan et al., 2017). Therefore, the middle meatus does not accurately capture the CRS sinus microbiome.

Although variation does exist between sinuses within an individual, no consistent differences were detected between different types of sinuses. In order to sample a representative microbial sinus community in CRS, our data supports the recommendation to sample in at least one site, but preferably more, within the sinus cavities.

## Conclusion

We have identified an association of OTUs from the genera *Corynebacterium* and *Dolosigranulum* with non-CRS sinuses and an increase in the genus *Escherichia* in CRS. The middle meatus alone does not provide a representative sample of the CRS sinus microbiota, as differences in taxa abundance identified in the sinus cavities between groups were not detected from middle meatus samples. Future studies of the sinus microbiota should include samples from at least one, and ideally multiple sinuses to obtain a more accurate understanding of the sinus microbiome in CRS.

## Author contributions

EC: carried out experiments, analyzed data, wrote and edited the manuscript; KL: recruited patients, collected samples, edited the manuscript; RC: analyzed data, wrote and edited the manuscript; JK: collected samples, designed experiments, edited the manuscript; MF and YN: designed experiments, collected samples, edited the manuscript; BO: designed experiments, wrote and edited the manuscript; JS and SW: designed analysis experiments, wrote and edited the manuscript; CB: designed the study, designed experiments, analyzed data, wrote and edited the manuscript; NS: designed the study, designed experiments, collected samples, wrote and edited the manuscript.

### Conflict of interest statement

The authors declare that the research was conducted in the absence of any commercial or financial relationships that could be construed as a potential conflict of interest.

## Footnotes

^1^arb-silva.de/download/arb-files

^2^http://qiita.microbio.me
