# Supplementary material for: Chronic Rhinosinusitis: Potential Role of Microbial Dysbiosis and Recommendations for Sampling Sites
Source: Front Cell Infect Microbiol. 2018 Feb 28;8:57. doi: 10.3389/fcimb.2018.00057 (PMC5836553; doi:10.3389/fcimb.2018.00057)
Supplement: Supplementary file 1 [file DataSheet1.DOCX]

**Table S1:** **Primers used for PCR Stage 1.** Primer names are indicated in the first column, and different regions of the primers are indicated separated into columns for ease of viewing.

| ***Primer name*** | ***Partial Illumina adaptor*** | ***phaser*** | ***16S priming region*** |
| --- | --- | --- | --- |
| Forward phaser0 | GTGACTGGAGTTCAGACGTGTGCTCTTCCGATCT |  | ACTCCTACGGGAGGCAGCAG |
| Forward phaser1 | GTGACTGGAGTTCAGACGTGTGCTCTTCCGATCT | T | ACTCCTACGGGAGGCAGCAG |
| Forward phaser2 | GTGACTGGAGTTCAGACGTGTGCTCTTCCGATCT | GT | ACTCCTACGGGAGGCAGCAG |
| Forward phaser3a | GTGACTGGAGTTCAGACGTGTGCTCTTCCGATCT | CGG | ACTCCTACGGGAGGCAGCAG |
| Reverse phaser0 | ACACTCTTTCCCTACACGACGCTCTTCCGATCT |  | GGACTACHVGGGTWTCTAAT |
| Reverse phaser1 | ACACTCTTTCCCTACACGACGCTCTTCCGATCT | A | GGACTACHVGGGTWTCTAAT |
| Reverse phaser2a | ACACTCTTTCCCTACACGACGCTCTTCCGATCT | TA | GGACTACHVGGGTWTCTAAT |
| Reverse phaser3 | ACACTCTTTCCCTACACGACGCTCTTCCGATCT | CTT | GGACTACHVGGGTWTCTAAT |

**Table S2: Primers used for PCR Stage 2.** Primer names are indicated in the first column, and different regions of the primers are indicated separated into columns for ease of viewing.

| ***Primer name*** | ***Illumina flow cell region*** | ***index*** | ***partial Illumina adaptor overlap*** |
| --- | --- | --- | --- |
| Enrichment_i7_1 | CAAGCAGAAGACGGCATACGAGAT | TCGCCTTA | GTGACTGGAGTTCAGACGTG |
| Enrichment_i7_2 | CAAGCAGAAGACGGCATACGAGAT | CTAGTACG | GTGACTGGAGTTCAGACGTG |
| Enrichment_i7_3 | CAAGCAGAAGACGGCATACGAGAT | TTCTGCCT | GTGACTGGAGTTCAGACGTG |
| Enrichment_i7_4 | CAAGCAGAAGACGGCATACGAGAT | GCTCAGGA | GTGACTGGAGTTCAGACGTG |
| Enrichment_i7_5 | CAAGCAGAAGACGGCATACGAGAT | AGGAGTCC | GTGACTGGAGTTCAGACGTG |
| Enrichment_i7_6 | CAAGCAGAAGACGGCATACGAGAT | CATGCCTA | GTGACTGGAGTTCAGACGTG |
| Enrichment_i7_7 | CAAGCAGAAGACGGCATACGAGAT | GTAGAGAG | GTGACTGGAGTTCAGACGTG |
| Enrichment_i7_8 | CAAGCAGAAGACGGCATACGAGAT | CCTCTCTG | GTGACTGGAGTTCAGACGTG |
| Enrichment_i7_9 | CAAGCAGAAGACGGCATACGAGAT | AGCGTAGC | GTGACTGGAGTTCAGACGTG |
| Enrichment_i7_10 | CAAGCAGAAGACGGCATACGAGAT | CAGCCTCG | GTGACTGGAGTTCAGACGTG |
| Enrichment_i7_11 | CAAGCAGAAGACGGCATACGAGAT | TGCCTCTT | GTGACTGGAGTTCAGACGTG |
| Enrichment_i7_12 | CAAGCAGAAGACGGCATACGAGAT | TCCTCTAC | GTGACTGGAGTTCAGACGTG |
| Enrichment_i7_14 | CAAGCAGAAGACGGCATACGAGAT | TCATGAGC | GTGACTGGAGTTCAGACGTG |
| Enrichment_i7_15 | CAAGCAGAAGACGGCATACGAGAT | CCTGAGAT | GTGACTGGAGTTCAGACGTG |
| Enrichment_i7_16 | CAAGCAGAAGACGGCATACGAGAT | TAGCGAGT | GTGACTGGAGTTCAGACGTG |
| Enrichment_i5_1 | AATGATACGGCGACCACCGAGATCT | TAGATCGC | ACACTCTTTCCCTACACGA |
| Enrichment_i5_2 | AATGATACGGCGACCACCGAGATCT | CTCTCTAT | ACACTCTTTCCCTACACGA |
| Enrichment_i5_3 | AATGATACGGCGACCACCGAGATCT | TATCCTCT | ACACTCTTTCCCTACACGA |
| Enrichment_i5_5 | AATGATACGGCGACCACCGAGATCT | GTAAGGAG | ACACTCTTTCCCTACACGA |
| Enrichment_i5_6 | AATGATACGGCGACCACCGAGATCT | ACTGCATA | ACACTCTTTCCCTACACGA |
| Enrichment_i5_7 | AATGATACGGCGACCACCGAGATCT | AAGGAGTA | ACACTCTTTCCCTACACGA |
| Enrichment_i5_8 | AATGATACGGCGACCACCGAGATCT | CTAAGCCT | ACACTCTTTCCCTACACGA |
| Enrichment_i5_11 | AATGATACGGCGACCACCGAGATCT | TCTCTCCG | ACACTCTTTCCCTACACGA |
| Enrichment_i5_12 | AATGATACGGCGACCACCGAGATCT | TCGACTAG | ACACTCTTTCCCTACACGA |
| Enrichment_i5_15 | AATGATACGGCGACCACCGAGATCT | TTCTAGCT | ACACTCTTTCCCTACACGA |
| Enrichment_i5_16 | AATGATACGGCGACCACCGAGATCT | CCTAGAGT | ACACTCTTTCCCTACACGA |
| Enrichment_i5_17 | AATGATACGGCGACCACCGAGATCT | GCGTAAGA | ACACTCTTTCCCTACACGA |
| Enrichment_i5_18 | AATGATACGGCGACCACCGAGATCT | CTATTAAG | ACACTCTTTCCCTACACGA |
| Enrichment_i5_20 | AATGATACGGCGACCACCGAGATCT | AAGGCTAT | ACACTCTTTCCCTACACGA |
| Enrichment_i5_21 | AATGATACGGCGACCACCGAGATCT | GAGCCTTA | ACACTCTTTCCCTACACGA |
| Enrichment_i5_22 | AATGATACGGCGACCACCGAGATCT | TTATGCGA | ACACTCTTTCCCTACACGA |

**Table S3:**  Patient characteristics. Patient data was collected via patient completed surveys at the time of consent. NC indicates data was not collected. N indicates no and Y indicates yes. In the case of smoking status, N = never, Y indicates smoker where Y1 = 1-10 per day, Y2 = 11-20 per day, Y3 = +20 per day, X indicates previously a smoker where X1 = < 1 year since smoking, X2 = 1-5 since smoking, X3 = > 5 years since smoking. The SNOT22 score is a measure of symptom severity which ranges from 1 - 22, where 1 is the least severe and 22 is the most.

| **Patient** | **Group** | **Age** | **Gender** | **Smoker** | **Asthma** | **SNOT22** |
| --- | --- | --- | --- | --- | --- | --- |
| 11 | CONTROL | 66 | M | N | N | 1 |
| 19 | CONTROL | 65 | M | Y1 | Y | 2 |
| 20 | CONTROL | 61 | M | N | N | 23 |
| 21 | CONTROL | 40 | F | NC | NC | 9 |
| 24 | CONTROL | 77 | M | NC | NC | NC |
| 31 | CONTROL | 50 | M | N | N | 8 |
| 41 | CONTROL | 74 | M | N | N | NC |
| 45 | CONTROL | NC | M | N | N | 8 |
| 47 | CONTROL | NC | M | n | N | 31 |
| 49 | CONTROL | NC | M | n | N | 18 |
| 50 | CONTROL | 45 | F | N | N | 3 |
| 999 | CONTROL | 69 | M | X3 | N | 10 |
| 1 | CRS | 66 | F | N | N | 19 |
| 2 | CRS | 43 | M | N | N | 34 |
| 4 | CRS | 31 | M | N | N | 81 |
| 5 | CRS | 38 | M | N | N | 65 |
| 6 | CRS | 58 | F | N | N | 37 |
| 7 | CRS | 61 | m | Y1 | N | 53 |
| 8 | CRS | 67 | M | N | N | 30 |
| 9 | CRS | 69 | M | X3 | Y | 39 |
| 10 | CRS | 45 | M | N | Y | 40 |
| 12 | CRS | 43 | F | X3 | Y | 58 |
| 13 | CRS | 24 | F | N | N | 35 |
| 14 | CRS | 53 | M | Y1 | N | 77 |
| 17 | CRS | 58 | F | N | Y | 34 |
| 23 | CRS | 28 | M | N | N | 85 |
| 26 | CRS | 47 | M | N | Y | 62 |
| 27 | CRS | 33 | M | N | Y | 38 |
| 28 | CRS | 47 | F | N | N | 64 |
| 42 | CRS | 38 | F | X2 | N | 72 |
| 43 | CRS | 49 | M | N | N | 42 |
| 44 | CRS | 33 | M | N | N | 23 |
| 51 | CRS | 26 | M | X2 | Y | 65 |

**Table S4.** Samples collected and processed from each patient. L and R indicate left or right, FRO is frontal, ETH is ethmoid, SPH is sphenoid, MAX is maxillary, NOST is right nostril, MM is middel meatus.

| **Patient** | **Health Status** | **LFRO** | **RFRO** | **LETH** | **RETH** | **LSPH** | **RSPH** | **LMAX** | **RMAX** | **NOST** | **LMM** | **RMM** |
| --- | --- | --- | --- | --- | --- | --- | --- | --- | --- | --- | --- | --- |
| **11** | Control | N | N | N | N | Y | Y | N | N | Y | Y | Y |
| **19** | Control | Y | Y | Y | Y | Y | Y | Y | Y | N | Y | Y |
| **21** | Control | N | N | Y | Y | N | N | N | N | N | N | N |
| **24** | Control | N | N | N | N | Y | Y | N | N | Y | Y | N |
| **31** | Control | N | N | N | N | Y | Y | N | N | Y | Y | N |
| **999** | Control | N | N | N | N | Y | Y | N | N | Y | Y | Y |
| **20** | Control | N | N | N | N | Y | Y | N | N | Y | Y | Y |
| **41** | Control | N | N | N | N | Y | Y | N | N | Y | N | Y |
| **45** | Control | N | N | N | N | Y | N | N | N | N | Y | N |
| **47** | Control | N | N | N | N | Y | Y | N | N | Y | Y | Y |
| **49** | Control | N | N | N | N | Y | Y | N | N | Y | Y | Y |
| **50** | Control | Y | N | Y | N | Y | N | Y | N | Y | Y | Y |
| **1** | CRS | Y | Y | Y | Y | Y | Y | Y | Y | Y | Y | Y |
| **2** | CRS | Y | Y | Y | Y | Y | Y | Y | Y | Y | Y | Y |
| **4** | CRS | Y | Y | Y | Y | Y | Y | Y | Y | Y | Y | Y |
| **5** | CRS | Y | Y | Y | Y | Y | Y | Y | Y | Y | Y | Y |
| **6** | CRS | Y | Y | Y | Y | Y | Y | Y | Y | Y | Y | Y |
| **7** | CRs | Y | Y | Y | Y | Y | Y | Y | Y | Y | Y | Y |
| **8** | CRS | Y | Y | Y | Y | Y | Y | Y | Y | Y | Y | Y |
| **9** | CRS | Y | Y | Y | Y | Y | Y | Y | Y | Y | Y | Y |
| **10** | CRS | Y | Y | Y | Y | Y | Y | Y | Y | N | Y | Y |
| **12** | CRS | Y | Y | Y | Y | Y | Y | Y | Y | Y | Y | Y |
| **13** | CRS | Y | Y | Y | Y | Y | Y | Y | Y | Y | Y | Y |
| **14** | CRS | Y | Y | Y | Y | Y | Y | Y | Y | Y | Y | Y |
| **17** | CRS | Y | Y | Y | Y | Y | Y | Y | Y | Y | Y | Y |
| **23** | CRS | Y | Y | Y | Y | Y | Y | Y | Y | N | Y | Y |
| **26** | CRS | Y | Y | Y | Y | Y | Y | Y | Y | Y | Y | Y |
| **27** | CRS | Y | Y | Y | Y | Y | Y | Y | Y | Y | Y | Y |
| **28** | CRS | Y | Y | Y | Y | Y | Y | Y | Y | Y | Y | Y |
| **42** | CRS | Y | Y | Y | Y | Y | Y | Y | Y | Y | Y | Y |
| **43** | CRS | Y | Y | Y | Y | Y | Y | Y | Y | Y | Y | Y |
| **44** | CRS | Y | Y | Y | Y | Y | Y | Y | Y | Y | Y | Y |
| **51** | CRS | Y | Y | Y | Y | Y | Y | Y | Y | Y | Y | Y |
