# Supplementary material for: Chronic Rhinosinusitis: Potential Role of Microbial Dysbiosis and Recommendations for Sampling Sites
Source: Front Cell Infect Microbiol. 2018 Feb 28;8:57. doi: 10.3389/fcimb.2018.00057 (PMC5836553; doi:10.3389/fcimb.2018.00057)
Supplement: Supplementary file 2 [file DataSheet2.docx]

**Supplementary Figure Legends**

**Figure S1.** Detected taxa in the mock community (Zymobiomics), with log2 fold change compared to expected relative abdunance. Data was fro two seperate sequencing runs, indicated in pink and blue. Taxa detection was determined by the best BLAST hit to a database of 16S rRNA genes of the mock community members.

**Figure S2.** Network analysis showing negative and positive correlations of the most highly correlated OTUs to factors. Networks were calculated using extended local similarity analysis and plotted using the Cytoscape3 software package.  Nodes are colored by genus taxonomic assignment, and metadata category nodes are colored red.  Glow behind nodes indicates the relationship to disease status (blue = positive correlation, red = negative).  Black lines indicate positive correlations, and the length indicates the strength (short = strong, long = weak).  Red lines indicate negative correlations and the length indicates the strength in the opposite direction (long = strong, short = weak).

**Figure S3.** Prevalence and relative abundance of anaerobes in CRS sinuses. Anaerobic genera were assigned manually, and the relative abundance in each sinus calculated from rarefied counts.

**Figure S4.** Prevalence and relative abundance of anaerobes in control sinuses. Anaerobic genera were assigned manually, and the relative abundance in each sinus calculated from rarefied counts.

**Figure S5.** Linear regression of genera found to be significnatly correlated to SNOT-22 scores (Spearman’s, FDR adjusted p<0.05). OTU numbers are indicated in grey boxes above each plot, and the genus taxonomic assignments are overlayed on each plot. Individaul data points are coloured by patient.
