# Supplementary figures and images for: Chronic Rhinosinusitis: Potential Role of Microbial Dysbiosis and Recommendations for Sampling Sites
Source: Front Cell Infect Microbiol. 2018 Feb 28;8:57. doi: 10.3389/fcimb.2018.00057 (PMC5836553; doi:10.3389/fcimb.2018.00057)

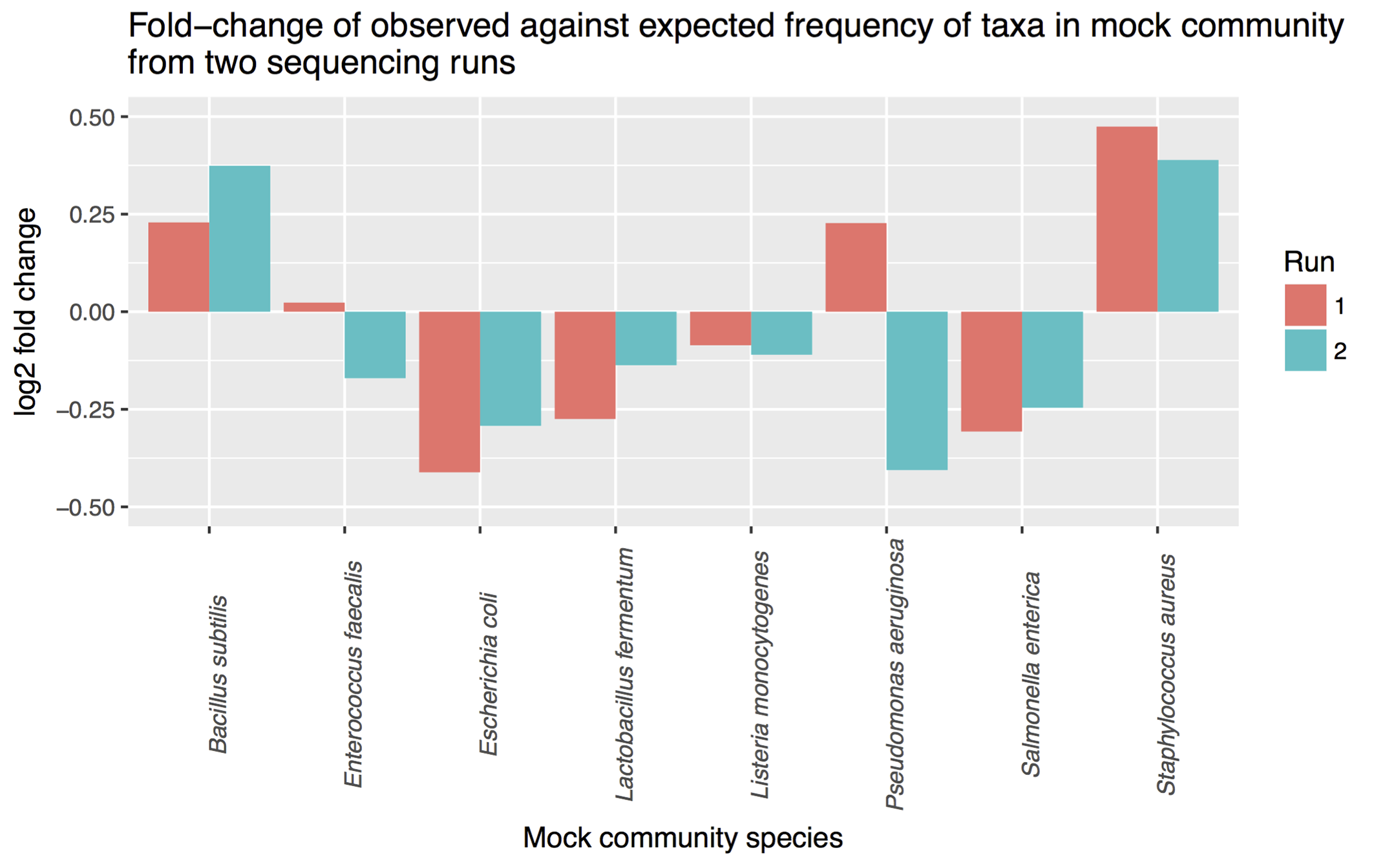

Supplement: Supplementary file 3 [file Image1.TIFF]

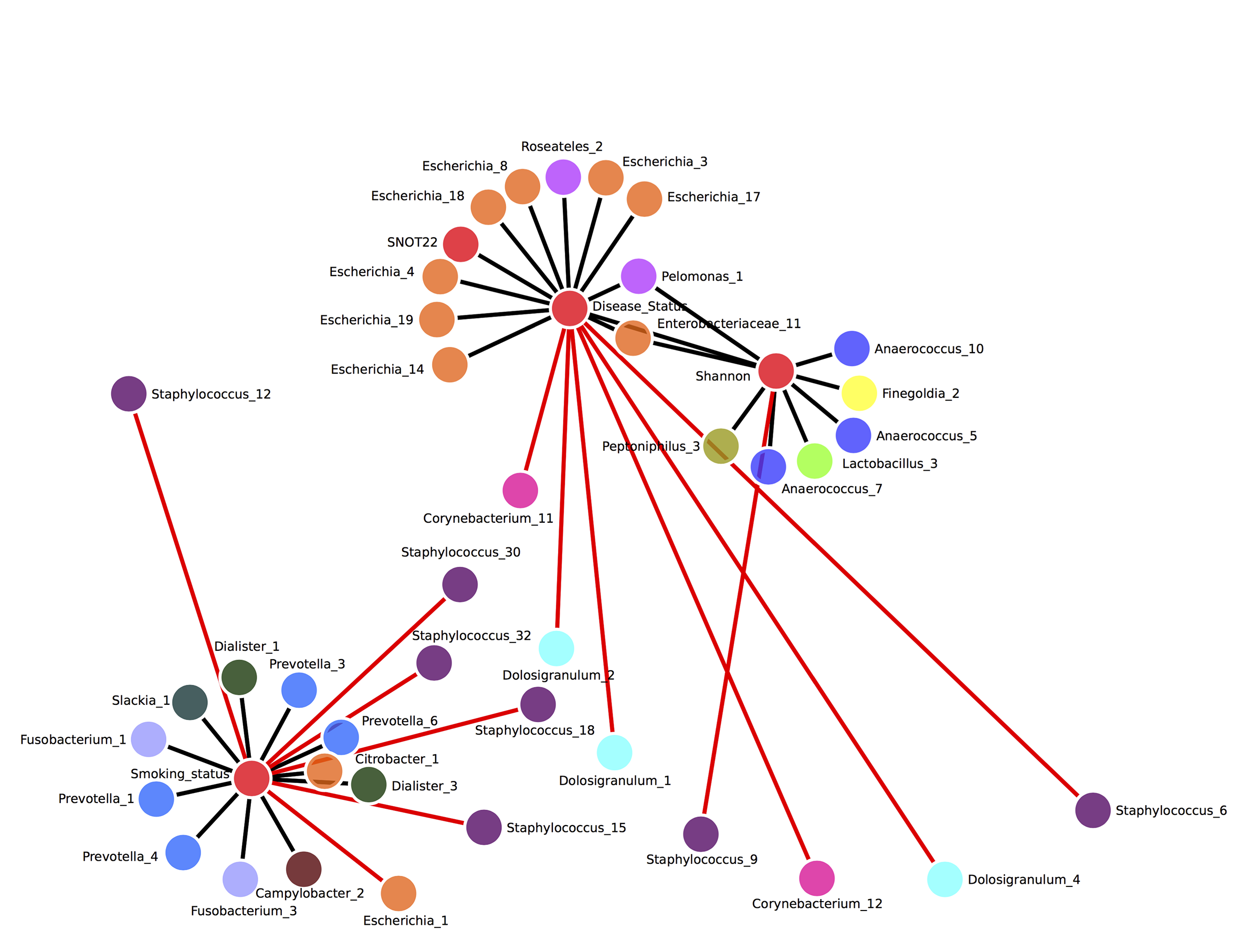

Supplement: Supplementary file 4 [file Image2.TIFF]

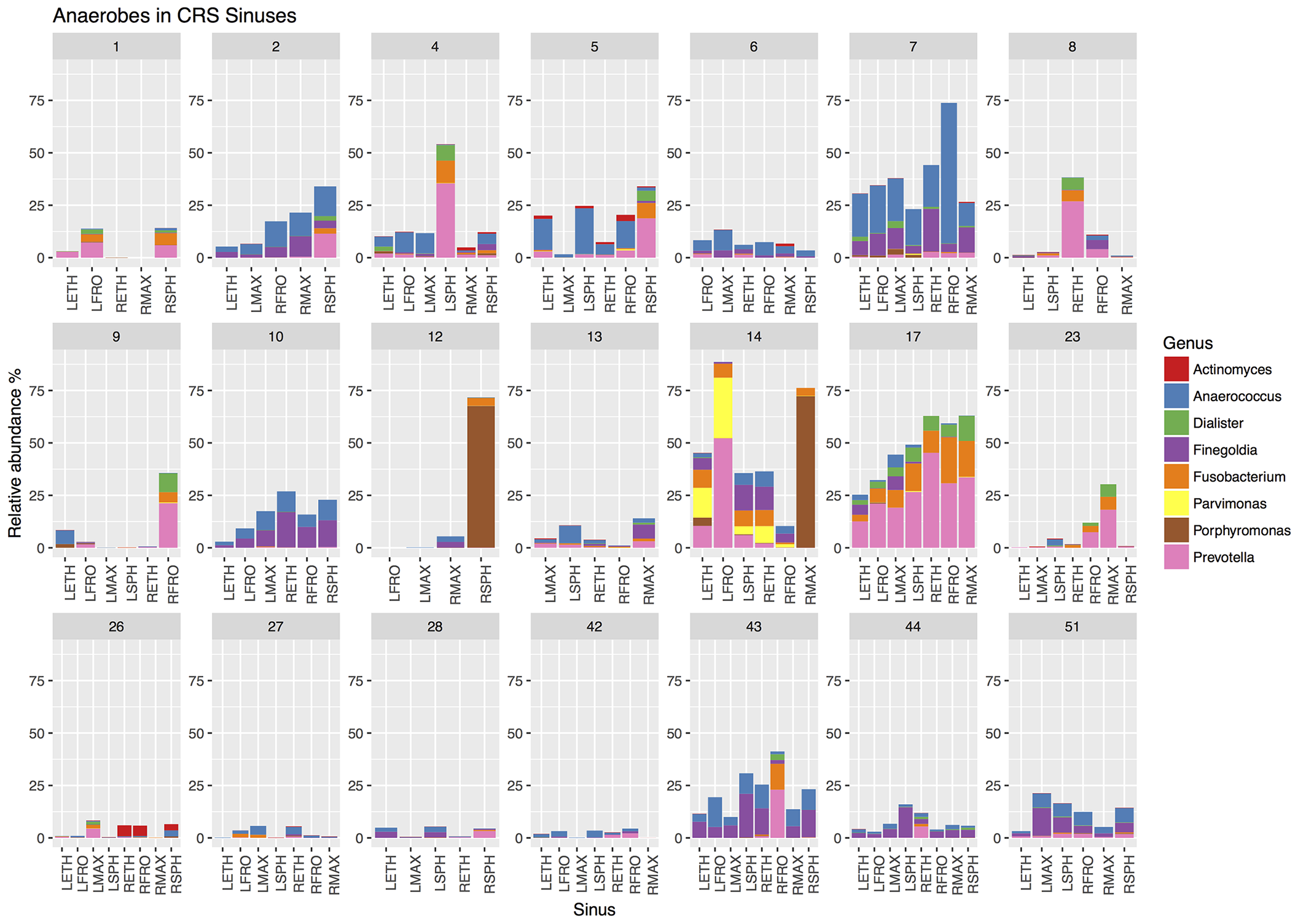

Supplement: Supplementary file 5 [file Image3.TIFF]

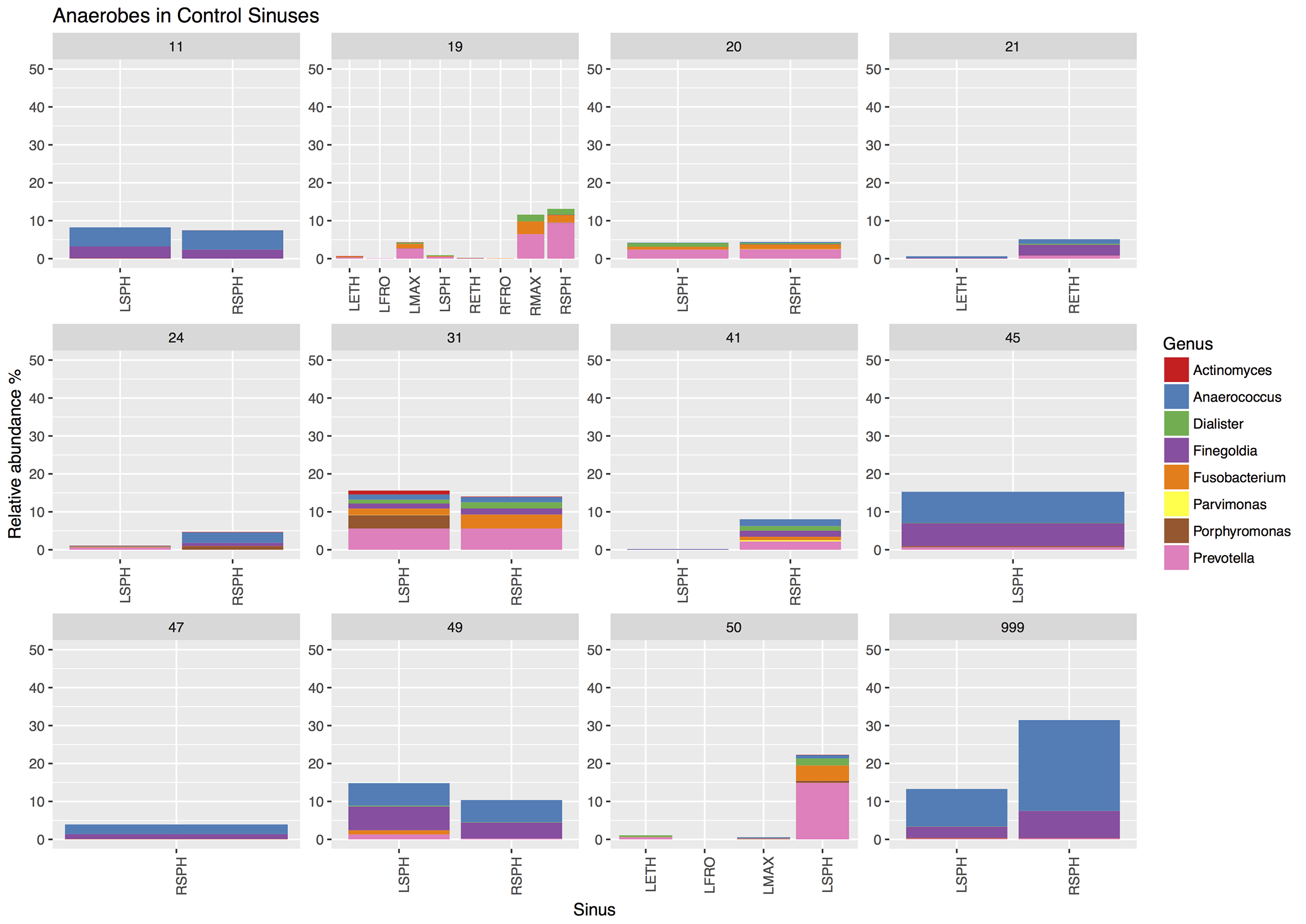

Supplement: Supplementary file 6 [file Image4.TIFF]

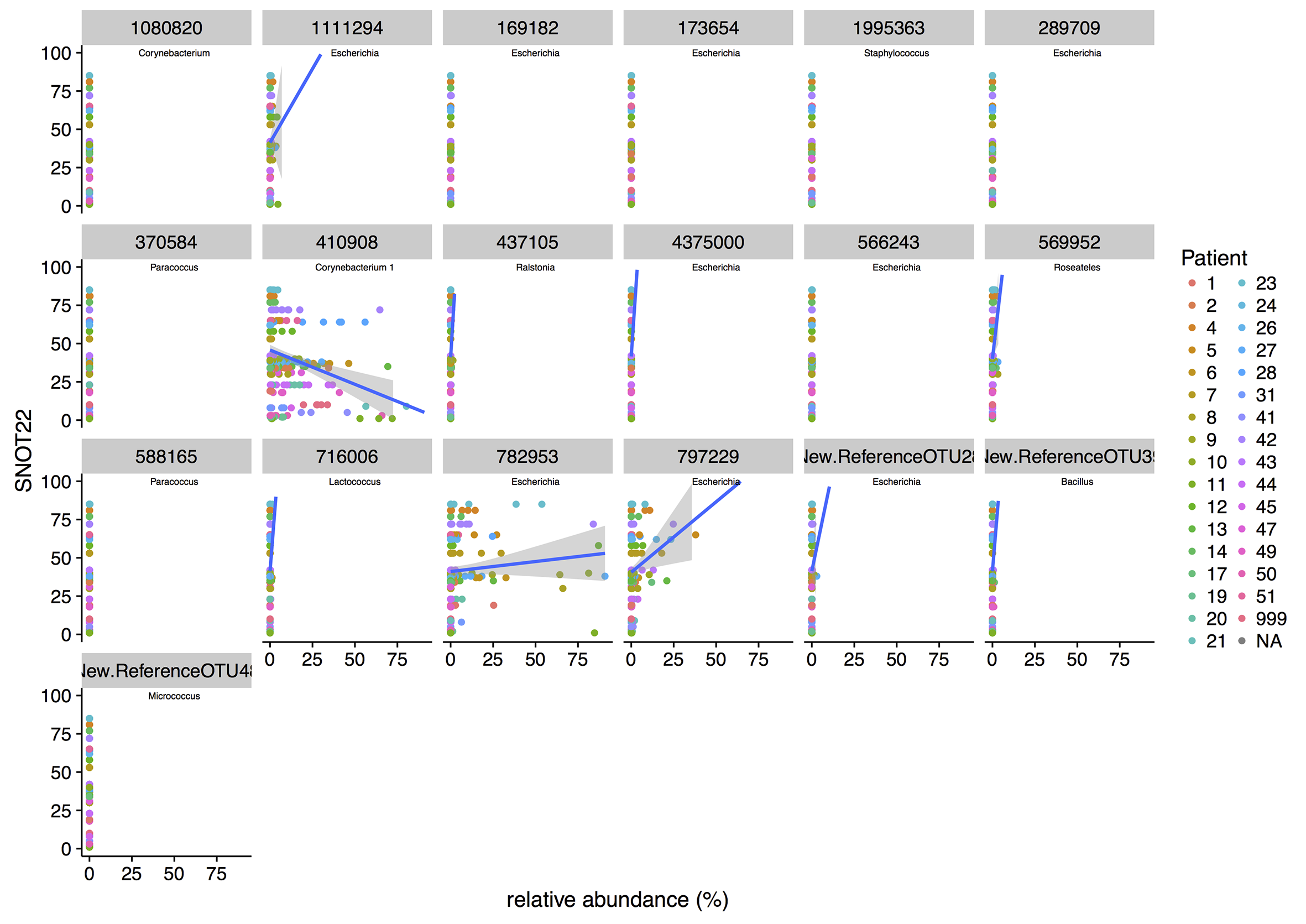

Supplement: Supplementary file 7 [file Image5.TIFF]
